# Supplementary material for: Conformal Coating of Stem Cell-Derived Islets for β Cell Replacement in Type 1 Diabetes
Source: Stem Cell Reports. 2019 Dec 12;14(1):91–104. doi: 10.1016/j.stemcr.2019.11.004 (PMC6962554; doi:10.1016/j.stemcr.2019.11.004)
Supplement: Document S1. Figure S1 [file mmc1.pdf]

Stem Cell Reports, Volume *14*

Supplemental Information

Conformal Coating of Stem Cell-Derived Islets for β Cell Replacement in Type 1 Diabetes

Aaron A. Stock, Vita Manzoli, Teresa De Toni, Maria M. Abreu, Yeh-Chuin Poh, Lillian Ye, Adam Roose, Felicia W. Pagliuca, Chris Thanos, Camillo Ricordi, and Alice A. Tomei

Supplemental Figure and legend

Figure S1:

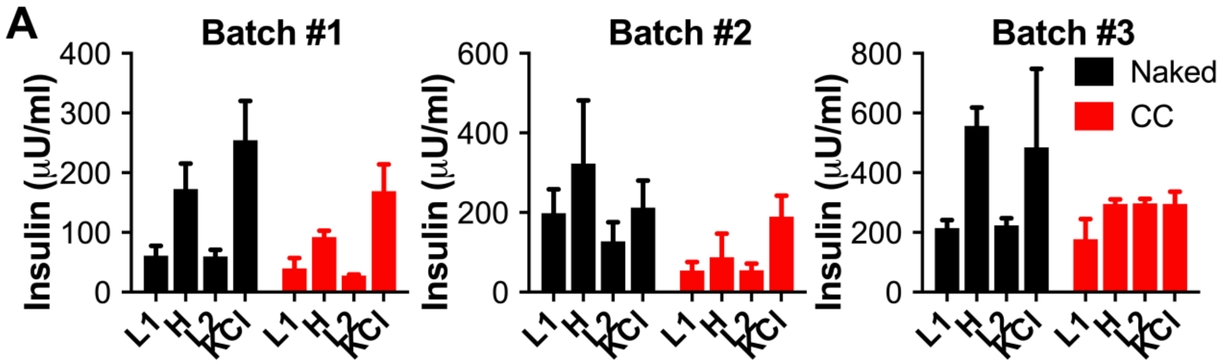

Figure S1: ***In vitro*** **GSIS assessment of unencapsulated and CC SC-islets**

(A) GSIS functionality of three independent batches of unencapsulated (black, naked) and conformal coated (red, CC) SC-islets 48 hours (S6d11) after encapsulation as absolute insulin secretion (n=3 wells assayed per condition) during sequential stimulation with 2.8mM glucose (L), 20mM glucose (H), 2.8mM glucose (L), and 30mM KCl.
