# Supplementary material for: Conformal Coating of Stem Cell-Derived Islets for β Cell Replacement in Type 1 Diabetes
Source: Stem Cell Reports. 2019 Dec 12;14(1):91–104. doi: 10.1016/j.stemcr.2019.11.004 (PMC6962554; doi:10.1016/j.stemcr.2019.11.004)
Supplement: Document S2. Article plus Supplemental Information [file mmc2.pdf]

Stem Cell Reports
Article
OPEN ACCESS

Conformal Coating of Stem Cell-Derived Islets for β Cell Replacement in Type 1 Diabetes

Aaron A. Stock,[1,2,7] Vita Manzoli,[1,7] Teresa De Toni,[1,2] Maria M. Abreu,[1] Yeh-Chuin Poh,[3] Lillian Ye,[3] Adam Roose,[3] Felicia W. Pagliuca,[3] Chris Thanos,[3] Camillo Ricordi,[1,2,4,5,6] and Alice A. Tomei[1,2,4,*]

[1]Diabetes Research Institute, University of Miami Miller School of Medicine, 1450 NW 10th Avenue, Miami, FL 33136, USA
[2]Department of Biomedical Engineering, University of Miami, Miami, FL 33146, USA
[3]Semma Therapeutics, Inc., Cambridge, MA 02142, USA
[4]Department of Surgery, University of Miami Miller School of Medicine, Miami, FL 33136, USA
[5]Department of Microbiology and Immunology, University of Miami Miller School of Medicine, Miami, FL 33136, USA
[6]Department of Medicine, University of Miami Miller School of Medicine, Miami, FL 33136, USA
[7]Co-first author
*Correspondence: atomei@miami.edu
https://doi.org/10.1016/j.stemcr.2019.11.004

SUMMARY

The scarcity of donors and need for immunosuppression limit pancreatic islet transplantation to a few patients with labile type 1 diabetes. Transplantation of encapsulated stem cell-derived islets (SC islets) might extend the applicability of islet transplantation to a larger cohort of patients. Transplantation of conformal-coated islets into a confined well-vascularized site allows long-term diabetes reversal in fully MHC-mismatched diabetic mice without immunosuppression. Here, we demonstrated that human SC islets reaggregated from cryopreserved cells display glucose-stimulated insulin secretion *in vitro*. Importantly, we showed that conformally coated SC islets displayed comparable *in vitro* function with unencapsulated SC islets, with conformal coating permitting physiological insulin secretion. Transplantation of SC islets into the gonadal fat pad of diabetic NOD-scid mice revealed that both unencapsulated and conformal-coated SC islets could reverse diabetes and maintain human-level euglycemia for more than 80 days. Overall, these results provide support for further evaluation of safety and efficacy of conformal-coated SC islets in larger species.

INTRODUCTION

Type 1 diabetes (T1D) is an autoimmune disease involving T cell-mediated destruction of insulin-secreting β cells in the pancreas, leading to impaired blood glucose regulation (Atkinson et al., 2014). More than a million children and adolescents were estimated to have T1D globally in 2017 (International Diabetes Federation, 2017). Despite sustained scientific progress in the field, most patients still rely on exogenous insulin to manage T1D (Redondo et al., 2018; Skyler, 2018). In the most severe cases, asymptomatic hypoglycemia can result in coma or death. Transplantation of insulin-secreting cells is indicated in individuals with labile T1D to replenish the insulin source and restore glucose homeostasis (Dean et al., 2017; Schuetz and Markmann, 2016; Shapiro et al., 2017). The islet transplantation field has grown tremendously in the past decades (Hering et al., 2016). Currently, two of the major limiting factors of pancreatic islet transplantation are the scarcity of donors and the need for life-long immunosuppression to prevent islet rejection and recurrence of autoimmunity (Stegall et al., 1996).

Various alternative cell sources are being explored (Orive et al., 2018), such as pig islets (Krishnan et al., 2017; Markmann et al., 2016; Salama and Korbutt, 2017) and stem cell-derived insulin-producing cell clusters (D'Amour et al., 2006; Millman and Pagliuca, 2017; Tomei et al., 2015). The latter could provide an unlimited source for replacement therapy. Although very promising, the use of alternative sources would not eliminate the need for immunosuppression.

Encapsulation may prevent immune rejection of transplanted islets and eliminate the need for immunosuppression (Korsgren, 2017). The goal of islet encapsulation is to provide cells with a physical semi-permeable barrier that prevents infiltration of immune cells while allowing smaller molecules such as oxygen, nutrients, glucose, and insulin to diffuse freely through the capsule. Despite efforts so far, encapsulated islets have failed to maintain physiological insulin secretion *in vitro* (Buchwald et al., 2018) and grant long-term insulin independence in the clinical setting (Calafiore and Basta, 2014; Shimoda and Matsumoto, 2017; Vaithilingam et al., 2017).

Our group recently reported a novel encapsulation method based on conformal coating via a fluidic device that minimizes capsule thickness, allowing physiological insulin secretion *in vitro* (Tomei et al., 2014). When transplanted in confined and well-vascularized sites, conformal-coated islets successfully maintained long-term euglycemia in a fully major histocompatibility complex (MHC)-mismatched allogeneic transplantation model in mice without immunosuppression (Manzoli et al., 2018). The present study aims to apply the conformal-coating platform to stem cell-derived islets (SC islets)

This is an open access article under the CC BY license (http://creativecommons.org/licenses/by/4.0/).

generated through a previously established protocol (Pagliuca et al., 2014), using clinically translatable encapsulation materials to demonstrate safety and efficacy of this β cell replacement strategy *in vitro* and *in vivo* in an immunodeficient mouse model.

RESULTS

Human SC Islets Reaggregated from Cryopreserved Cells Are Functional *In Vitro*

Cryorecovered stage-6 cells (S6d1) were aggregated into SC islets in spinner flasks for 2–10 days (Figure 1A) and displayed an overall yield of 42% at day 3 (S6d4), 24% at day 5 (S6d6), and 21% at day 8 (S6d8) post thawing (Figure 1B), but comparable viability of about 75% (Figures 1C and 1D). Importantly, the fraction of mature β cells (NKX6.1$^+$ C-peptide$^+$) in SC islets was found to increase during S6 reaggregation from 12.7% to 36.1% (cold-shipped batches) and from 19.2% to 33.8% (cryoshipped batches) compared with end-of-stage-5 (S5) (Figure 1E). Characterization of SC islet functionality during reaggregation revealed that dithizone (DTZ) staining was weaker in S6d7 cells (d6 reaggregation) (Figure 1F), which corresponded to lower glucose-stimulated insulin secretion (GSIS) functionality compared with S6d11 (Figure 1G), although GSIS indexes and deltas were comparable (Figure 1H).

Overall, stage-6 SC islets reaggregated from cryopreserved cells (n = 6 batches) contained 19.6%–48.8% mature β cells (NKX6.1$^+$ C-peptide$^+$) and a high level of endocrine population with >90% chromogranin A positivity (CHGA$^+$) (Figure 1I and Table 1). SC islets were functional *in vitro* as assessed by GSIS, with a mean stimulation index of 3.6 (Figure 1J and Table 1). We concluded that SC islet reaggregation from cryopreserved cells increases the purity of glucose-sensing and insulin-secreting cells.

Conformal-Coated SC Islets Perform Similarly to Unencapsulated SC Islets *In Vitro*

To assess the feasibility of conformal coating of SC islets, we performed encapsulation of S6d7–S6d9 SC islets using our proprietary fluidic platform (Manzoli et al., 2018; Tomei et al., 2014) using clinically applicable hydrogels made of 5% poly(ethylene glycol)-maleimide (PEG-MAL) crosslinked with 2 kDa dithiolated PEG (SH-PEG-SH) and supplemented with NEP peptide and PEG-oligoethylene sulfide (OES) nanofibrils. We then cultured conformal-coated (CC) SC islets in parallel with non-encapsulated ones *in vitro* for up to 7 days (S6d14). After encapsulation, CC SC islets stained for DTZ more weakly than primary islets, although this was no less intense than unencapsulated SC islets (Figure 2A). CC SC islets appeared overall viable up to 7 days during *in vitro* culture, although higher cell death was observed on the external cell layers of CC SC islets as compared with unencapsulated cells (Figure 2B). Oxygen consumption rates were comparable between CC and unencapsulated SC islets (Figure 2C), suggesting that live cells in CC SC islets are as metabolically active as unencapsulated cells. Importantly, we found that conformal coatings were generally complete (Figure 2D) and the average thickness was 25.45 μm (±11.43 μm) (Figure 2E).

Static GSIS assessment of three different batches of SC islets revealed that absolute values of secreted insulin of CC SC islets were lower compared with unencapsulated SC islets (Figure S1A), although no statistically significant differences were found in the GSIS index or delta at 24 and 48 h post coating (Figure 2F). We concluded that the static GSIS profile of CC SC islets was comparable with that of unencapsulated SC islets *in vitro* up to 7 days of culture after coating (S6d14), although absolute quantities of secreted insulin were diminished.

Different Encapsulation Methods Yield Comparable SC Islet Functionality *In Vitro*

Next, we analyzed whether the conformal-coating process was adversely affecting SC islets compared with other traditional microencapsulation procedures using alginate (ALG) and PEG. We performed encapsulation experiments via our conformal-coating platform where (1) both 8-arm PEG-MAL and PEG-SH crosslinker (Xlinker) were added to SC islets in Hank's balanced salt solution (HBSS) (CC, Figure 3B), (2) only 8-arm PEG-MAL without Xlinker was added to SC islets in HBSS to prevent PEG gelation (no Xlinker, Figure 3C) in order to evaluate potential toxicity of coating materials, and (3) only HBSS was added to SC islets (HBSS, Figure 3D) to evaluate potential negative effects of shear forces due to the coating process. In parallel, we microencapsulated SC islets using an electrostatic droplet generator with alginate (micro(ALG), Figure 3E) or a combination of alginate and PEG (micro(PEGALG), Figure 3F) that we previously demonstrated confers higher *in vivo* functionality to islet allografts in the intraperitoneal cavity than ALG (Villa et al., 2017). Quantification of z stacks of live/dead confocal images revealed that CC islets exhibited a viability of 79%, which was comparable with naked SC islets and with SC islets encapsulated in traditional capsules (Figure 3G). These results suggest lack of shear-induced cell death and limited toxicity of the coating polymers. Central necrosis was visible in SC islets encapsulated in ALG and PEGALG larger microcapsules (Figures 3E and 3F).

Absolute GSIS insulin secretion and delta were lower for all encapsulated conditions compared with unencapsulated SC islets, but similar among all encapsulated groups

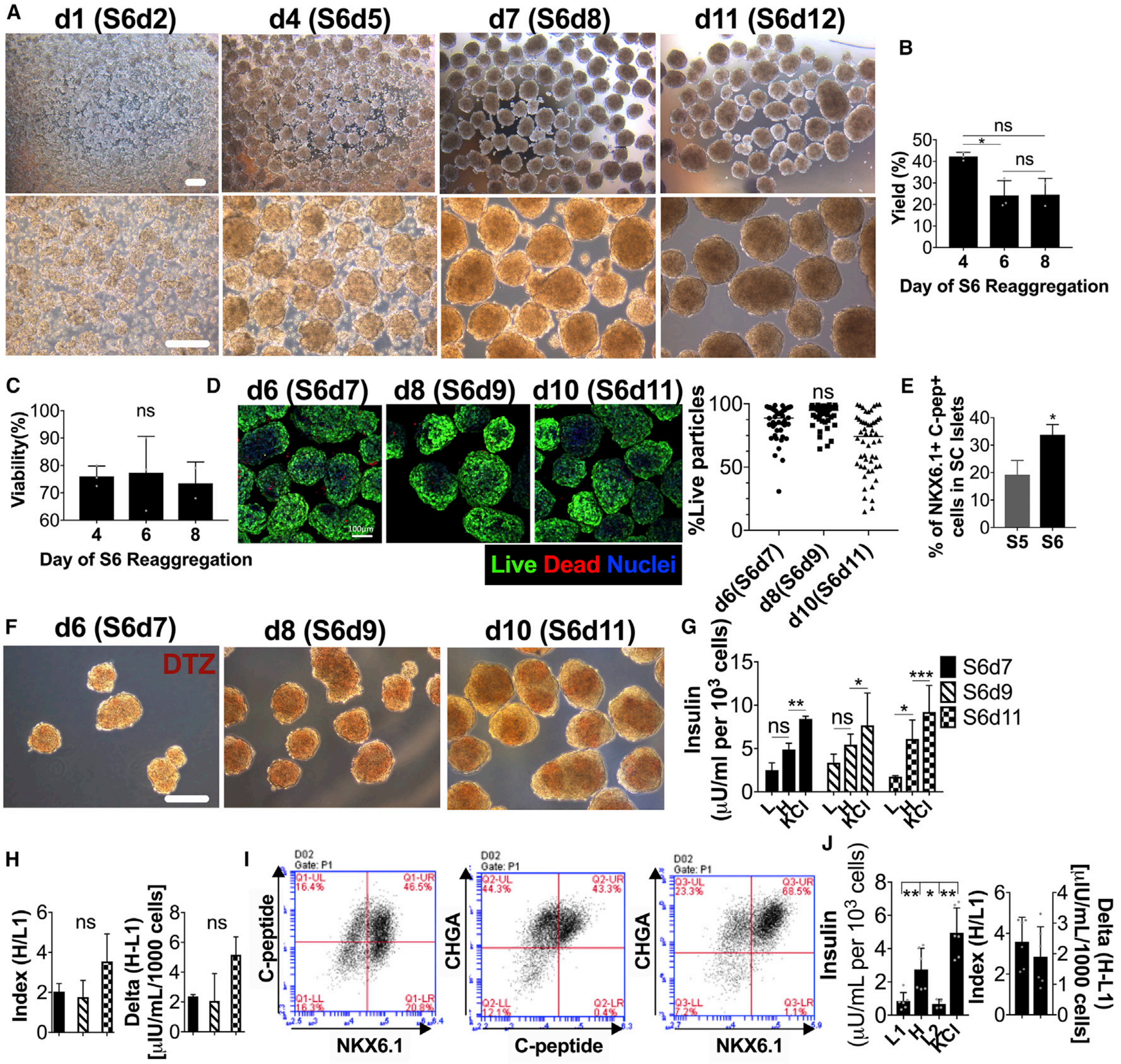

Figure 1. *In Vitro* Assessment of Stage-6 SC Islets Reaggregated from Cryopreserved End-of-Stage-5 Cells

(A–C) Phase-contrast images (A) of stage-6 day-1 SC cells thawed and reaggregated in spinner flasks for 1 (S6d2) to 11 (S6d12) days at different magnifications. Scale bars, 200 μm. Live cell yield (B) and viability (C) of SC islets post thawing during reaggregation in spinner flasks (n = 3 reaggregation batches) assessed using trypan blue exclusion and automated cell counting.

(D) Confocal images (maximal projection of 150 μm-thick z stacks) and quantification of live/dead stained stage-6 day-7 (S6d7), S6d9, and S6d11 after thawing and reaggregation. Scale bar, 100 μm.

(E) β cell purity in SC islets as percentage of NKX6.1$^+$C-peptide$^+$ cells at the end of S6 reaggregation compared with the end of S5 before cryopreservation (n = 3 differentiation batches).

(F–H) Dithizone (DTZ) staining (F) and static GSIS functionality (G and H) of S6d7, S6d9, and S6d11 SC islets as GSIS absolute insulin secretion (G), index and delta (H) (n = 3 wells per condition assayed). SC islets were stimulated sequentially with 2.8 mM glucose (L), 20 mM glucose (H), and 30 mM KCl solutions. Scale bar, 200 μm.

(I and J) Characterization of six independent differentiation batches of S6 SC islets reaggregated from cryopreserved S5 cells assessed by flow cytometry (I) and by GSIS (J) (sequential stimulation with 2.8 mM glucose [L], 20 mM glucose [H], 2.8 mM glucose [L], and 30 mM KCl solutions). $^*p < 0.05$; $^{**}p < 0.01$; $^{***}p < 0.01$. ns, no significant differences found.

All error bars are derived from standard deviations.

(Figure 3H). However, the stimulation index of CC SC islets was comparable with that of unencapsulated SC islets and SC islets encapsulated in micro(ALG) and micro(PEGALG).

We concluded that encapsulation was minimally affecting viability and GSIS functionality of SC islets, although the kinetics of insulin secretion appear to be

Table 1. Characterization of Six Batches of SC Islets Differentiated from Research Line HuES8 Cells by Semma Therapeutics before Cryopreservation and Reaggregation and Used in This Study

	GSIS					Flow Cytometry		
Differentiation #	GSIS Day	LG Insulin Secretion µIU/mL/1,000 Cells	HG Insulin Secretion	KCl Insulin Secretion	Stimulation Index (HG/LG)	$NKX6.1^{+}$ $C\text{-}Peptide^{+}$ (%)	$CHGA^{+}$ (%)	Day of Stage 6
1	S6d6	0.34	1.57	3.49	4.64	46.5	90.2	S6d2
2	S6d5	0.79	1.67	6.62	2.11	19.6	84.5	S6d8
3	S6d9	1.8	4.05	4.73	2.25	31.9	96.9	S6d5
4	S6d7	0.51	1.12	3.7	2.17	29.7	97.5	S6d8
5	S6d6	0.75	3.4	4.76	4.54	21.8	98.6	S6d9
6	S6d8	0.9	4.21	6.8	4.66	48.8	97.8	S6d9

Glucose-stimulated insulin secretion (GSIS) and flow cytometry were used for characterization.

attenuated by hydrogel microencapsulation independently of the encapsulation method. Unlike what was previously shown for human islets encapsulated in large ALG microcapsules (Buchwald et al., 2018), we found that dynamic GSIS of CC SC islets was comparable to unencapsulated SC islets, with no delays in insulin secretion of CC SC islets compared with unencapsulated SC islets (Figure 3I).

Conformal-Coated SC Islets Perform Similarly to Unencapsulated SC Islets *In Vivo*

Following *in vitro* studies demonstrating suitable viability and functionality, we performed *in vivo* studies to determine the ability of unencapsulated and CC SC islets to restore normoglycemia in the NOD-scid immunodeficient mouse model compared with primary human islets. Because the volume of conformal-coated SC islets was comparable with that of unencapsulated SC islets, we could transplant CC SC islets in a confined site such as the fat pad and were not restricted to implantation in the intraperitoneal space, as would be the case with traditional ALG microcapsules (Vegas et al., 2016). Thus, we transplanted human islets (HI), unencapsulated SC islets, and CC SC islets in the mammary (MFP) or the epididymal (EFP) fat pad of chemically-induced diabetic female and male NOD-scid mice, respectively, at different doses (1.25k, 2k, and 2.5k IEQ) using a clinically-applicable resorbable biological scaffold (Berman et al., 2016). Because functionality of unencapsulated and CC SC islets before implantation was comparable (Figure 2), the same dose of unencapsulated and CC SC islets was transplanted.

First, we compared recipients of comparable doses of unencapsulated SC islets and unencapsulated HI and found that primary HI recipients that reversed diabetes after islet transplantation displayed better overall glycemic control immediately after transplantation than SC islet recipients (Figures 4A and 4B). However, only at the highest transplant dose (2.5k IEQ/mouse, $p = 0.007$) did HI show faster engraftment, which resulted in earlier reversal (6/7; median reversal time [MRT], 1 day) than SC islets (6/8; MRT, 58 days). Diabetes reversal rates were comparable when 2k IEQ/mouse ($p = 0.12$; HI: 5/5; MRT, 2.5 days; SC islets: 2/2; MRT, 32 days) or 1.25k IEQ/mouse ($p = 0.16$; HI: 3/5; MRT, 12 days; SC islets: 3/5; MRT, 83 days) were transplanted (Figure 4C). The lowest cell dose (1.25k IEQ/mouse) was able to reverse diabetes only in 60% of subjects when either HI or SC islets were transplanted. Blood glucose control at 78–102 days after transplant of 2.5k and 2k IEQ/mouse doses in animals that reversed diabetes after transplantation was comparable between groups and regulated at human levels (2.5k IEQ HI: 91 ± 12 mg/dL; 2k IEQ HI: 77 ± 16 mg/dL; 1.25k IEQ HI: 116 ± 19 mg/dL; 2.5k IEQ SC islets: 118 ± 80 mg/dL; 2k IEQ SC islets: 58 ± 25 mg/dL; 1.25k IEQ SC islets: 246 ± 162 mg/dL). Glucose tolerance of 2.5k and 2k IEQ permouse doses of HI and SC islet recipients after long-term (>100 days) implantation assessed via intraperitoneal glucose tolerance test (IPGTT) was comparable, suggesting very efficient glucose clearance, while glucose tolerance of 1.25 IEQ/mouse HI was impaired (Figures 4D and 4E; $p = 0.019$).

We then compared unencapsulated to CC SC islets and found a similar performance *in vivo* in terms of glycemic control of animals that reversed diabetes after transplantation (Figure 4F). We compared two different doses, 2k and 2.5k IEQ/mouse, since at lower doses unencapsulated SC islets were unable to reverse diabetes consistently. CC SC islets (2.5k IEQ: 4/5; MRT, 29 days; 2k IEQ: 4/5; MRT, 39 days)

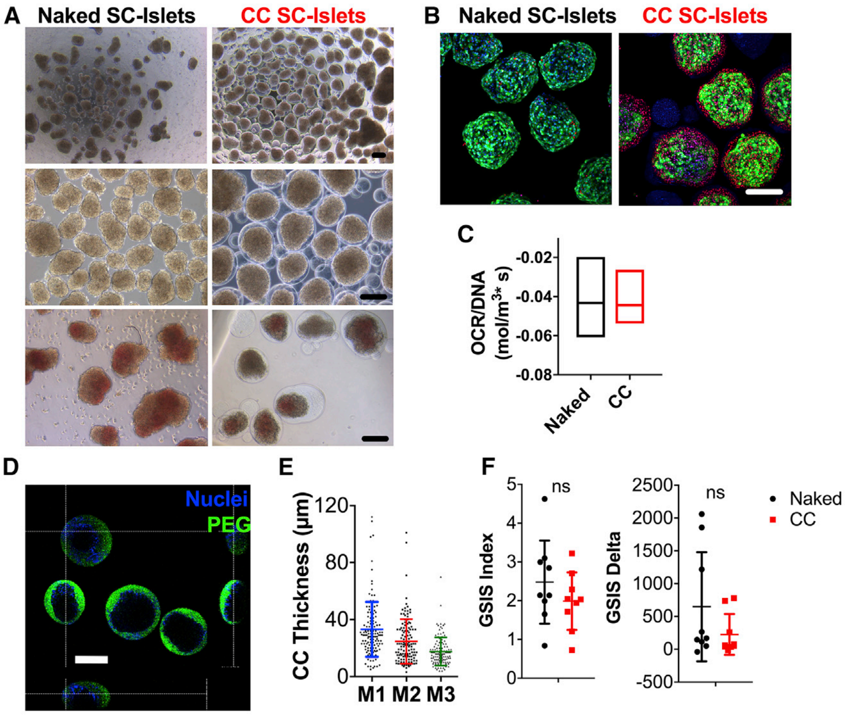

Figure 2. *In Vitro* Assessment of Unencapsulated and CC SC Islets

(A) Phase-contrast images of unencapsulated (left, naked) and conformal-coated (right, CC) S6d9 SC islets at different magnifications (top and center) and stained with dithizone (DTZ, bottom). Scale bars, 200 μm.

(B) Live/dead images (maximal projection of 150-μm-thick z stacks) of naked (left) and CC (right) SC islets 48 h (S6d11) after coating as confocal images of live (green)/dead (red) stained cells (nuclei: blue). Scale bar, 100 μm.

(C) Oxygen consumption rates (OCR) of naked (black) and CC (red) S6d11 SC islets normalized for DNA content.

(D) Capsule completeness as orthogonal projections of confocal images of anti-PEG (green) stained capsules. Nuclei: blue. Scale bar, 200 μm.

(E) Capsule thickness quantification (n = 130 CC islets evaluated) on three axes (M1, M2, M3).

(F) GSIS functionality of naked (black) and CC (red) SC islets 48 h (S6d11) after encapsulation as GSIS index and delta (n = 3 independent batches of naked and CC SC islets and n = 3 well repeats assayed per batch) during sequential stimulation with 2.8 mM glucose (L), 20 mM glucose (H), 2.8 mM glucose (L), and 30 mM KCl solutions. ns, no significant differences found.

All error bars are derived from standard deviations.

showed a trend toward improved diabetes reversal rate compared with unencapsulated SC islets at both doses analyzed, although not significantly (Figure 4G; 2.5k IEQ p = 0.12; 2k IEQ p = 0.60). Blood glucose control at 78–102 days after transplant was comparable between unencapsulated and CC SC islets and regulated at human levels (2.5k IEQ CC SC islets: 60 ± 12 mg/dL; 2k IEQ CC SC islets: 58 ± 11). Glucose tolerance of unencapsulated and CC SC islets recipients after long-term (>100 days) implantation assessed via IPGTT was comparable (Figures 4J and 4K; p = 0.23) and suggested very efficient glucose clearance. Return to hyperglycemia after retrieval of HI, unencapsulated and CC SC islets grafts confirmed that diabetes correction was due to graft function.

Graft-wide histological analysis of unencapsulated and CC HI and SC islet grafts revealed the presence of INS^+ β cells, GLU^+ α cells, and INS^+GLU^+ polyhormonal (PH) cells (Figures 5A–5F). No significant difference in the prevalence of polyhormonal cells (Figure 5B) was found in naked (NK) and CC SC islet grafts that were explanted either less than 30 days after transplant (POD <30) or at least 100 days after transplantation (POD >100) in the fat pad of diabetic NOD-scid compared with HI grafts. Despite not reaching statistical significance, it should be noted that there was a general trend of decrease in the number of PH cells as a fraction of all insulin-positive cells in grafts explanted at POD >100 compared with those explanted at POD <30.

Importantly, we found that islet architecture and composition was preserved in CC SC islet grafts at POD >100 (Figure 5F), since no significant differences in the ratio of INS^+ to all INS^+ or GLU^+ cells were detected in naked or CC human or SC islet grafts at early or late time points (Figure 5C). These results suggest that the phenotype of SC islets was stable in conformal capsules throughout the implantation period of more than 100 days.

Macrophages were present in explanted grafts of all conditions. No significant differences in the number of macrophages ($MAC2^+$ cells) per unit area of tissue analyzed were detected between naked or CC human or SC islets (Figure 5G). Concordantly, no significant differences in relative α-smooth muscle actin (αSMA) prevalence in arbitrary units per unit area of tissue analyzed was detected between naked or CC human or SC islets (Figure 5H), suggesting lack of capsule fibrosis.

We concluded that SC islets were able to reverse diabetes over the long term (>80 days) after implantation in confined vascularized sites in mice, and that encapsulation through conformal coating did not negatively affect and potentially even improved their *in vivo* function.

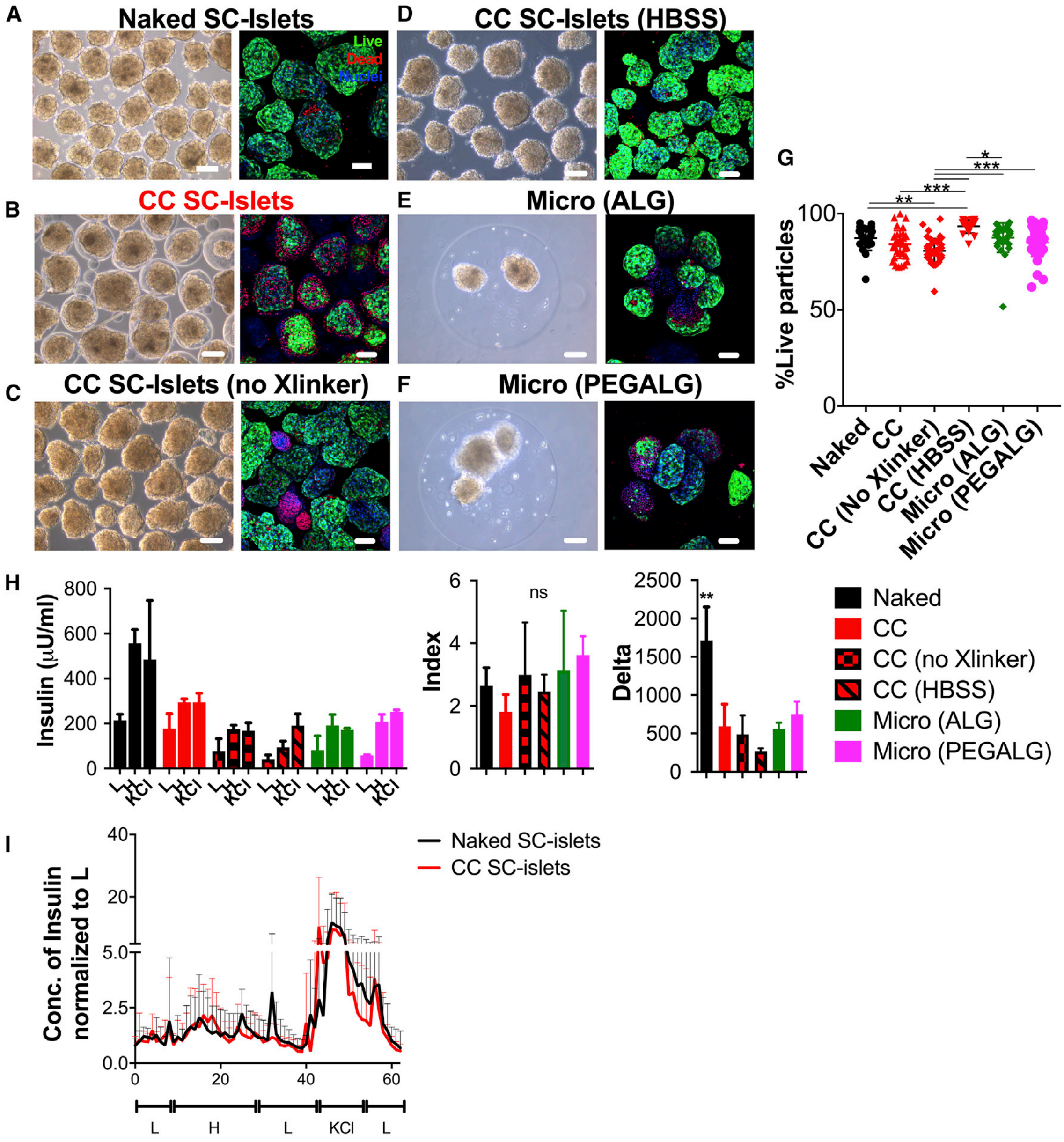

Figure 3. Encapsulation Methods Comparison *In Vitro*

(A–G) Phase-contrast images (left) and live/dead confocal images (maximal projection of 150-μm-thick z stacks) of live (green)/dead (red) stained cells (nuclei: blue) (right) and quantification (individual data points represent the percent viability for each z level imaged) (G) of unencapsulated (A, naked), CC (using PEG and SH-PEG-SH Xlinker as pre-gel solution to resuspend SC islets before running them through the CC process) (B), CC no Xlinker (using PEG without SH-PEG-SH as solution unable to form gels to resuspend SC islets before running them through the CC process) (C), CC HBSS (using HBSS as solution to resuspend SC islets before running them through the CC process) (D), 1.2% MVG alginate (ALG) microencapsulated using electrostatic droplet generators (E), and 1.2% MVG alginate and PEG (PEGALG) (F) microencapsulated SC islets. Scale bars, 200 μm.

(H) Static GSIS functionality of naked (black), CC (red), CC no Xlinker (red with black squares), CC HBSS (red with black stripes), ALG microencapsulated (green), and PEGALG microencapsulated (fuchsia) S6d9 SC islets as absolute insulin secretion, index, and delta during sequential stimulation with 2.8 mM glucose (L), 20 mM glucose (H), and 30 mM KCl solutions. $^{**}p < 0.01$ (n = 3 wells assayed per condition).

(I) Perifusion (100 μL/min perfusion with L: 2.7 mM glucose; with H: 20 mM glucose; with KCl: 2.7 mM glucose and 30 mM KCl solutions) of naked (black) and CC (red) SC islets normalized to L values. Four batches of naked and CC SC islets per condition. ns, no significant differences found.

All error bars are derived from standard deviations.

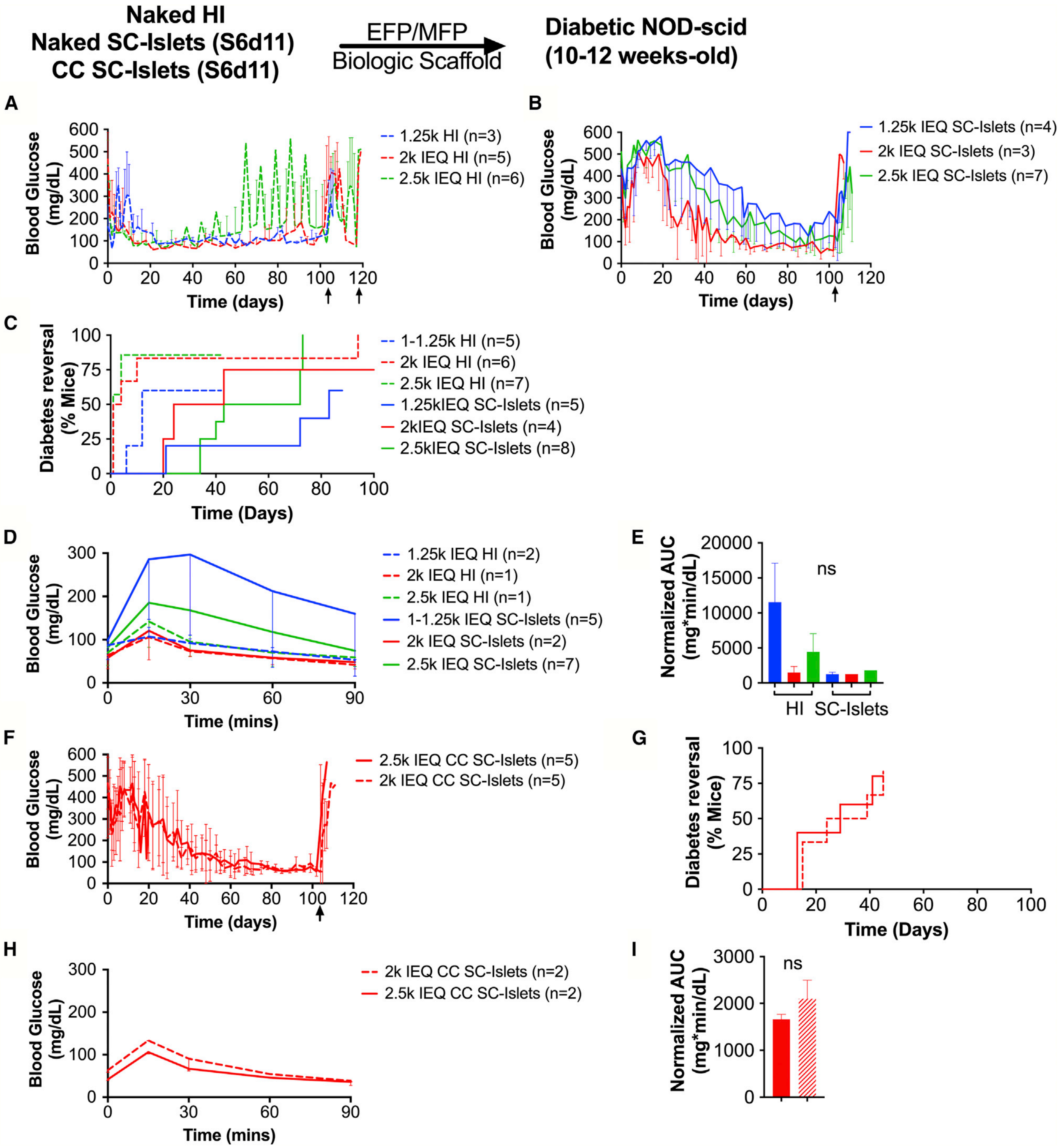

Figure 4. *In Vivo* Assessment of Unencapsulated and CC SC Islets Compared with Human Islets

(A–E) Average blood glucose (mg/dL) of diabetic NOD-scid mice that became normoglycemic after implantation of with 2.5k IEQ (green), 2k IEQ (red), or 1.25k IEQ (blue) HI (dashed line), unencapsulated HI (A, dashed line), or S6d11 SC islets (B, solid line) in the gonadal fat pad. Diabetes reversal efficiency (C) and glucose tolerance of recipient mice as average blood glucose during IPGTT (D) and area under the curve (AUC) derived from IPGTT (E) are shown.

(F–I) Average blood glucose of mice that became normoglycemic (F), diabetes reversal efficiency (G), and glucose tolerance of recipient mice as blood glucose during IPGTT (H); and AUC derived from IPGTT (I) of diabetic NOD-scid mice transplanted with 2.5k IEQ (solid line) or 2k IEQ (dashed line) CC (red) SC islets in the fat pad. ns, no significant differences found.

(legend continued on next page)

DISCUSSION

Transplantation of polymer-encapsulated islets has demonstrated strong efficacy in rodent models both in protecting encapsulated islets from the immune system and facilitating the diffusion of nutrients, glucose, and insulin through the microcapsule, resulting in glucose homeostasis (Chen et al., 2015). Frequently, however, these successes could not be recapitulated in large animal models such as non-human primates (O'Shea et al., 1984; Safley et al., 2018; Scharp and Marchetti, 2014), likely due to their bipedal locomotion leading to capsule displacement into the pelvis, and lower intraperitoneal oxygen tension below the threshold limit for islet function (Barkai et al., 2016).

Conformal coating seeks to overcome limitations of traditional microencapsulation—restriction to the intraperitoneal space and hypoxia-induced islet death (Dionne et al., 1993; Muthyala et al., 2017)—while thwarting the immune response to transplanted allogeneic tissue (Manzoli et al., 2018). Those benefits are all primarily the result of the relatively thin (approximately a few tens of micrometers) hydrogel coating on the islet surface that can be accomplished through conformal coating (Tomei et al., 2014). This contrasts with the thicker coatings (a few hundreds of micrometers) associated with traditional microencapsulation (Qayyum et al., 2017). The most obvious improvement derived from thinner coatings is a shorter diffusion path for molecules such as oxygen, glucose, and insulin. Increased access to oxygen is achieved, since an islet bearing a thinner coating would be in nearer proximity to a blood vessel (Duvillie, 2013) than an islet with a thicker coating; this would ameliorate the hypoxia-induced islet core death that is often associated with grafts in the intraperitoneal space (Safley et al., 2018).

The shorter diffusion path of the CC membrane that regulates inward glucose diffusion and outward insulin secretion may also result in better overall euglycemia maintenance. Accordingly, we observed no delay in the perifusion response of CC compared with unencapsulated SC islets. Conversely, microcapsules have shown delayed and blunted perifusion responses (Buchwald et al., 2018). Importantly, *in vivo* CC SC islets regulated glycemia at characteristic human levels (~100 mg/dL), whereas other groups transplanting microencapsulated SC islets achieved euglycemia on the higher end of the normal range (~200 mg/dL) (Vegas et al., 2016). In addition to the coating thickness, this observed difference may be the result of the rodent model, dosing, and transplant site disparities between the experiments as previously reported (Rodriguez-Diaz et al., 2018). Furthermore, glucose bolus clearance during IPGTT was found to be temporally equivalent between human and SC islets, as well as between unencapsulated and CC SC islets. Peak blood glucose concentration occurred within 15 min after glucose administration and did not exceed 200 mg/dL. This comports with peak glucose levels during oral glucose tolerance test in human subjects, and is in agreement with findings that human islets are engaged at lower glucose levels than native or transplanted mouse islets in mouse hosts and that it is the donor islet species that determines the glycemic set point (Abdul-Ghani et al., 2010; Merani et al., 2008; Nishimura et al., 2017; Rodriguez-Diaz et al., 2018; Schneider et al., 2005).

While CC SC islets maintain a comparable insulin secretion profile and stimulation index with those of unencapsulated islets, there is a notable diminution of the absolute quantities of insulin secreted by the former. Reduced insulin secretion was also observed in SC islets encapsulated using traditional methods. While unencapsulated SC islets grown in suspension can shed dead cells, hydrogel encapsulated islets are unable to, since those dead cells are immobilized within the capsules. Despite lower absolute insulin secretion in CC islets, comparable efficiency of reversing diabetes was observed after implantation in NOD-scid mice at comparable doses. The oxygen consumption rate has been shown to be predictive of the islet graft outcome (Papas et al., 2015), with poor oxygenation being a critical factor underlying islet encapsulation failure (Papas et al., 2016; Smith et al., 2017). We found that the *in vitro* oxygen consumption rate was similar between unencapsulated and encapsulated SC islets, suggesting that overall viability was not compromised by conformal coating. In larger animal models, reduced absolute insulin secretion in encapsulated SC islets may result in reduced efficacy of diabetes reversal. In the event this is observed, the dose of CC islets per animal may need to be increased, which is clinically feasible given that SC islets are an unlimited source.

Minimizing coating thickness is advantageous beyond its one-dimensional implications for diffusion because the overall graft volume scales as the cube of the change in capsule diameter. So, as the coating thickness decreases from those typical of microencapsulation to those typical

Average blood glucose at POD 78–102: 2.5k IEQ HI, 91 ± 12 mg/dL; 2k IEQ HI, 77 ± 16 mg/dL; 1.25k IEQ HI, 116 ± 19 mg/dL; 2.5k IEQ SC islets, 118 ± 80 mg/dL; 2k IEQ SC islets, 58 ± 25 mg/dL; 1.25k IEQ SC islets, 246 ± 162 mg/dL; 2.5k IEQ CC SC islets, 60 ± 12 mg/dL; 2k IEQ CC SC islets, 58 ± 11. Median reversal time: 2.5k IEQ HI, 1 day; 2k IEQ HI, 2.5 days; 1.25k IEQ HI, 12 days; 2.5k IEQ SC islets, 58 days; 2k IEQ SC islets, 32 days; 1.25k IEQ SC islets, 83 days; 2.5k IEQ CC SC islets, 29 days; 2k IEQ CC SC islets, 39 days; 2.5k IEQ CC SC islets, 21 days; 2k IEQ CC SC islets, 45 days.
All error bars are derived from standard deviations.

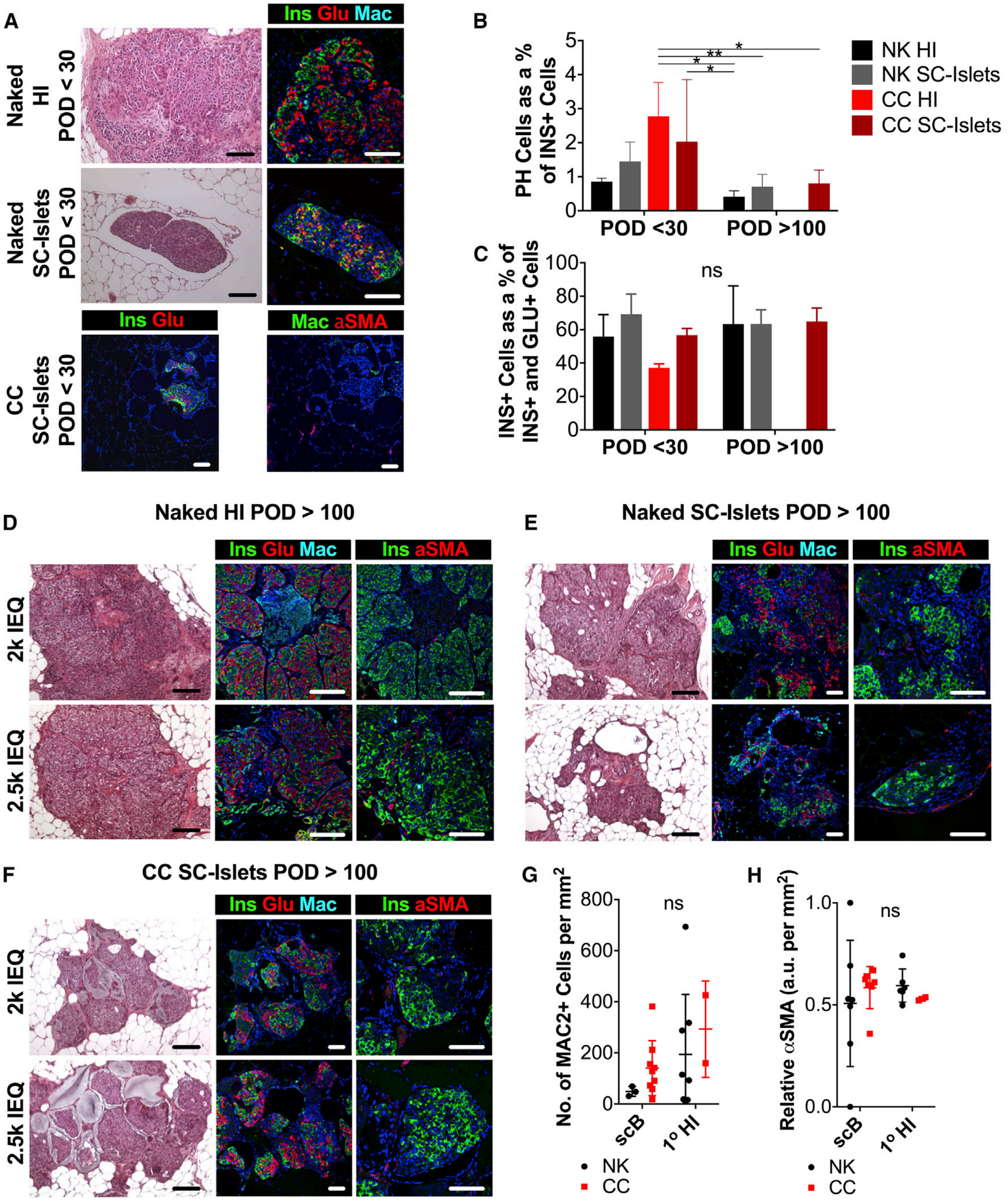

Figure 5. Histological Characterization of HI and SC Islet Grafts at Early and Late Time Points after Implantation in Mice

(A) Light-microscope images of H&E-stained (left panels) and confocal-microscope images (right panels) of 5-μm-thick sections of fat pad grafts of human islets (top), unencapsulated SC islets (middle, Naked), and CC SC islets (bottom) within 30 days after implantation (POD <30) in chemically diabetic STZ mice using resorbable biological scaffolds stained for insulin (green), glucagon (red), macrophages (naked, cyan; CC, green), α-smooth muscle actin (αSMA; CC, red) and nuclei (DAPI, blue).

(B and C) Quantification of $INS^{+}GLU^{+}$ polyhormonal (PH) cells as a percentage of all INS^{+} cells (B) and quantification of graft composition as percentage of INS^{+} β cells in all INS^{+} cells or GLU^{+} cells (C) for naked (NK) and CC HI and SC islet grafts at POD <30 and POD >100. ns, no significant difference found. NK HI: POD <30 n = 2, POD >100 n = 6; NK SC islets: POD <30 n = 3, POD >100 n = 7; CC HI: POD <30 n = 2, POD >100 n not evaluated; CC SC islets: POD <30 n = 4, POD >100 n = 8.

(legend continued on next page)

of conformal coating, the overall graft volume for a human dose of encapsulated islets decreases from about 1 L to about 10 mL, which is in the same magnitude order of the volume of unencapsulated islets. Minimizing graft volumes is necessary to allow implantation of SC islets in confined sites, which increases safety because of retrievability and possibility for graft monitoring, and improves *in vivo* functionality of microencapsulated insulin-producing cells (Villa et al., 2017).

While traditional encapsulation hydrogels are formed by ionic crosslinking of alginate with divalent cations such as Ca^{2+} (Lee and Mooney, 2012) and hydrogel stability may be compromised *in vivo* due to the presence of monovalent cations (Nunamaker et al., 2007), the conformal-coating hydrogel is based on Michael-type addition covalent crosslinking (Garcia, 2014; Kharkar et al., 2016; Tomei et al., 2014) and involves the formation of a new carbon-sulfur bond, which is degradable only at elevated temperature and high pH (Allen and Humphlett, 1966). We recently showed that PEG-based hydrogels and microcapsules exhibit more robust mechanical properties and stability than ALG-based hydrogels and microcapsules (Verheyen et al., 2019). For these reasons, we believe that PEG-based conformal coatings would exhibit increased temporal stability *in vivo* as compared with alginate-based microcapsules.

Increased patient access to islet transplantation would beget a new problem due to the limited supply of donor pancreata (Berney and Johnson, 2010). Advances in differentiation protocols have enabled the production of phenotypically stable and monohormonal insulin-secreting cells from embryonic or induced pluripotent stem cells (Pagliuca et al., 2014). In contrast to previous stem cell-derived insulin-secreting cell products requiring further differentiation *in vivo* after transplantation (Kroon et al., 2008), these SC islets are terminally differentiated at the time of transplantation and have shown efficacy in diabetes reversal *in vivo* in rodent models (Pagliuca et al., 2014; Vegas et al., 2016). Our work with SC islets, along with the work of others, has shown that SC islets can be used for transplantation at sites peripheral to that of their manufacture when they are shipped cold as clusters or shipped cryopreserved and reaggregated on site. One important point of consideration is the loss of cells during reaggregation culture (Figure 1B). While this may seem inefficient, others have reported on the value of this cell loss in eliminating irrelevant cell types and enriching the final cell population for endocrine cells (Veres et al., 2019). As such, purification by reaggregation is advantageous because fewer cells would be necessary to represent a curative transplanted dose, which would reduce the oxygen requirements of the graft and the concomitant cell loss in the immediate post-transplant period. Furthermore, as the safety of stem cell-derived products is established and evaluated, it is beneficial to limit the probability of adverse events, which is directly proportional to the transplanted cell number.

When transplanted in the gonadal fat pad of diabetic NOD-scid mice, CC SC islets performed similarly to unencapsulated SC islets in terms of glycemic control. Importantly, we observed less than 3% of polyhormonal cells in grafts that were explanted 100 days after transplantation, which supports that the cells did not undergo *in vivo* dedifferentiation, in accordance with what was previously reported (Pagliuca et al., 2014) and observed by other groups (Vegas et al., 2016). The timing of reversal of diabetes will likely be related to the potency of the cell line and protocol used, and the dose of cells provided, all of which are being optimized in future work.

In conclusion, for islet transplantation to have a meaningful impact on the T1D community, a renewable β cell source must be coupled with a robust immunoprotection platform. It has already been shown that microencapsulated SC islets can reverse diabetes long-term in immunocompetent mice (Vegas et al., 2016). Complementarily, we have shown that CC mouse islets can reverse diabetes long-term in a fully MHC-mismatched model (Manzoli et al., 2018). Given these promising findings, we believe that combining conformal coating and SC islets is a promising venture to achieving long-term diabetes reversal in fully MHC-mismatched diabetic recipients, and in this paper we have laid the groundwork by demonstrating efficacy of CC SC islets in an immunocompromised murine model of T1D.

(D–F) Light-microscope images of H&E-stained (left panels) and confocal-microscope images (right panels) of 5-μm-thick sections of fat pad grafts of naked human islets (D), naked SC islets (E), and CC SC islets (F) explanted at POD >100 stained for insulin (green), glucagon (red), macrophages (cyan), and nuclei (DAPI, blue) (left panel) and for insulin (green), αSMA (red), and nuclei (DAPI, blue) (right panel). Transplanted doses: top, 2k IEQ; bottom, 2.5k IEQ. Scale bars, 100 μm.
(G) Quantification of graft-infiltrating macrophages as $MAC2^{+}$ cells per square millimeter of graft tissue analyzed for naked (NK) and CC HI (naked: n = 8; CC: n = 2) and SC islet (naked: n = 3; CC: n = 9) grafts.
(H) Quantification of graft fibrosis as degree of αSMA prevalence in arbitrary units (a.u.) per square millimeter of graft tissue analyzed for naked (NK) and (CC) HI (naked: n = 8; CC: n = 2) and SC islets (naked: n = 3; CC: n = 9) grafts. All depicted scale bars represent 100 μ. All error bars are derived from standard deviations.

EXPERIMENTAL PROCEDURES

Differentiation and Culture of SC Islets

SC islets were provided by Semma Therapeutics, a wholly owned subsidiary of Vertex Pharmaceuticals (Boston, MA), and produced from the research-grade embryonic stem cell line HuES8, following the research differentiation protocol described in Millman et al. (2016), with two exceptions: LDN (BMP inhibitor) treatment was moved from stage 3 (S3) to S5, and S6 culture medium is as described below. SC islets were dissociated and cryopreserved at the end of S5 differentiation. After thawing, S6 d1 cells were reaggregated by suspension culture (ABLE Biott, distributed by Reprocell; Betsville, MD) at 60 rpm, 37°C, 5% CO_2, and 95% humidity in MCDB medium supplemented with 2% BSA and 1× GlutaMAX (Semma Therapeutics) (Pagliuca et al., 2014).

After thawing, S6 d1 cells were reaggregated into SC islets by suspension culture (ABLE Biott, distributed by Reprocell, Betsville, MD) at 60 rpm, 37°C, 5% CO_2, and 95% humidity in MCDB medium supplemented with 2% BSA and 1× GlutaMAX (Semma Therapeutics) (Pagliuca et al., 2014). SC islets were shipped to Miami either as S6d4-S6d8 reaggregated SC islets (cold-shipped batches) or as end-of-S5 cyropreserved cells and thawed and reaggregated in Miami (cryoshipped batches).

TrypLE Express Enzyme (Thermo Fisher Scientific, Waltham, MA) was used to dissociate SC islets into single-cell suspensions. Single-cell counts (or yields) and viability for post thaw and culture monitoring were determined using Gibco trypan blue stain (0.4%) exclusion (Thermo Fisher Scientific) and a TC20 Automated Cell Counter (Bio-Rad, Hercules, CA). For flow-cytometry characterization of cell differentiation, cell sample was fixed, blocked, and permeabilized with ICC buffer (5% donkey serum/0.05% Triton X-100 in PBS), incubated sequentially with primary antibodies: mouse anti-Nkx6.1 (DSHB, cat. #FSSA12), rat anti-C-pep (DSHB, cat. #GN-1D4-Sl), rabbit anti-CHGA (Abcam, Cambridge, UK, cat. #ab15160), rabbit anti-CDX2 (Abcam, cat. #ab76541), and rabbit anti-Sox9 (Epitomics, cat. #AC-0284RUOC); and secondary antibodies: anti-mouse AF488 (Thermo Fisher, cat. #A21202), anti-rat PE (Jackson Immunoresearch, cat. #712-116-150), anti-rabbit AF647 (Thermo Fisher, cat. #A31573), and anti-rabbit PE (Jackson Immunoresearch, cat. #711-116-152); and acquired using an Accuri C6 Plus (BD Biosciences, San Jose, CA). For gating, samples stained with secondary antibodies only were used as negative controls.

Encapsulation

Conformal Coating

A coating solution of 5% (v/w) 10-kDa 8-arm PEG (75% functionalized with maleimide groups) (Jenkem Technology, Plano, TX) supplemented with 0.5% (w/v) PepGel peptide (FLIVIGSII GPGGDGP) (New England Peptide, Gardner, MA) (NEP peptide) and 10 mg/mL PEG-oligoethylene sulfide (OES_5) nanofibers (synthesized as described in Brubaker et al., 2015), and crosslinked with homobifunctional 2-kDa SH-PEG-SH at a 3:1 molar ratio was used. SC islets were resuspended at a 50,000 IEQ per mL in coating solution at pH 3.5 and conformally coated as previously described (Tomei et al., 2014).

Microencapsulation

Microencapsulation of SC islets in 1.2% (w/v) ALG (UP-MVG, Novamatrix, Norway) or in 1.2% (w/v) ALG and 5% (v/w) PEG (PEGALG) was performed as previously described (Villa et al., 2017).

In Vitro Assessment of Viability and Functionality of Encapsulated SC Islets

Cluster viability was assessed by live/dead staining (Molecular Probes, Eugene, OR) and z-stack imaging with an Sp5 inverted confocal microscope (Leica Biosystems, Wetzlar, Germany). z-stack images were quantified using ImageJ (NIH, Bethesda, MD) software. Since PEG-encapsulated cells could not be liberated after coating, single-cell characterization of cell viability was not possible. Cluster oxygen consumption rate was also evaluated as previously described (Villa et al., 2017).

Functionality was assessed through static GSIS assay in 24-well plate-cell culture inserts (EMD Millipore, Temecula, CA) at a density of 500 IEQ per well in triplicate wells by sequential exposure with 2.8 mM glucose (L) for 1 h, 20 mM glucose (H) for 1 h, 2.8 mM glucose (L) for 1 h, and 30 mM KCl for 1 h. Dynamic GSIS was also investigated through perifusion assay as previously described (Tomei et al., 2014). In brief, cells were perifused (100 μL per min) sequentially with 2.8 mM glucose (L) for 8 min, 20 mM glucose (H) for 20 min, 2.8 mM glucose (L) for 15 min, 30 mM KCl for 10 min, and 2.8 mM glucose (L) for 8 min.

Capsule Thickness and Completeness

Capsule completeness was evaluated by immunostaining using biotinylated anti-PEG antibody (cat. #ab53449, Abcam), Alexa-Fluor488-conjugated streptavidin (Life Technologies, cat. #S11223), and confocal-microscope imaging (Leica Microsystems). Capsule thickness was quantified on phase-contrast images using ImageJ Software (NIH).

Mice, Diabetes Induction, Cluster Transplantation, and Graft Function Assessments

Female and male NOD-scid mice were used at 10–15 weeks of age as transplant recipients after being rendered diabetic following fasting and five daily intravenous injections of 50 mg/kg streptozotocin (Sigma-Aldrich, St. Louis, MO). Diabetic animals received 1.25k–2.5k IEQ of unencapsulated HI isolated by the University of Miami Diabetes Research Institute cGMP facility, or received unencapsulated or CC SC islets in the epididymal fat pad (EFP) or mammary fat pad (MFP), as previously described (Berman et al., 2016; Villa et al., 2017). In brief, SC islets were resuspended in NOD-scid autologous plasma, and human recombinant thrombin (Recothrom; Zymogenetics, Seattle, WA) was added to form a resorbable biological scaffold (Berman et al., 2016). Transplanted animal non-fasting blood glucose was monitored to determine diabetes reversal (three consecutive blood glucose readings <250 mg/dL).

An IPGTT was conducted on POD 100. In brief, mice were fasted 6 h prior to IPGTT and then injected intraperitoneally with 2 g per kg glucose. Blood glucose measurements were taken at 0, 10, 20, 30, 45, 60, 90, and 120 min after administration.

After >100 days (POD >100), animals were subjected to graftectomy, and subsequent diabetes reversal was monitored (three consecutive blood glucose readings >250 mg/dL).

All animal studies were performed under protocols reviewed and approved by the University of Miami Institutional Animal Care and Use Committee.

Graft Histology and Analysis

Animals were euthanized and grafts retrieved either within 30 days of implantation (POD <30) or at least 100 days after implantation (POD >100). Formalin-fixed grafts were embedded in paraffin, thin sectioned (5 μm in thickness), and processed for immunohistochemistry as previously described (Manzoli et al., 2018; Villa et al., 2017). Primary antibodies: insulin (Dako, cat. #A0564), glucagon (Biogenex, cat. #PU039-UP), CD31 (Abcam, cat. #ab28364), Mac2 (Cedarlane, cat. #CL8942AP), and aSMA-cy3 (Sigma, cat. #14–5773). Secondary antibodies (all from Molecular Probes): goat anti-guinea pig Alexa Fluor 488 (cat. #A11073), chicken anti-rabbit Alexa Fluor 594 (cat. #A21442), goat anti-guinea pig Alexa Fluor 647 (cat. #A21450), chicken anti-rabbit Alexa Fluor 488 (cat. #A21441), and chicken anti-rat Alexa Fluor 647 (cat. #A21472).

For graft-wide analysis of temporal phenotypic stability of transplanted SC islets, macrophage presence, and fibrotic deposition, a VS120 Virtual Slide Microscope (Olympus, Tokyo, Japan) was used to acquire immunofluorescence micrographs of whole graft slices. For analysis, a custom MATLAB (Mathworks, Natick, MA) routine was used to identify all nuclei from micrographs; all nuclei were then classified as INS^+GLU^+ polyhormonal cells (PH), INS^+GLU^- β cells, or INS^-GLU^+ α cells. For quantification of macrophage infiltration, a custom MATLAB routine was used to identify all nuclei from micrographs; all nuclei were then classified as either $MAC2^+$ or $MAC2^-$, and counts were normalized to the tissue area analyzed. For quantification of graft fibrosis, a custom MATLAB routine was used to quantify net fluorescence intensity corresponding to net fibrotic deposition in the form of αSMA staining (less that of CD31 to exclude αSMA staining of blood vessels and immune cells) and was normalized to the tissue area analyzed.

Statistical Analysis

Data are presented as means ± standard deviation unless otherwise noted. Statistical significance was determined using GraphPad Prism (GraphPad Software, La Jolla, CA) via either two-tailed Student's t test or one-way ANOVA, followed by Tukey's post hoc test. Survival data were analyzed using a log-rank Mantel-Cox test. A p value of less than 0.05 was considered significant.

SUPPLEMENTAL INFORMATION

Supplemental Information can be found online at https://doi.org/10.1016/j.stemcr.2019.11.004.

AUTHOR CONTRIBUTIONS

V.M. and A.A.S. wrote the manuscript and generated data. T.D.T. and M.M.A. generated data. Y.C.P., L.Y., A.R., F.W.P., and C.T. generated the SC islets and contributed to the design of the experiments. A.A.T. and C.R. conceived the project. A.A.T. designed the research and wrote the manuscript.

CONFLICTS OF INTEREST

A.A.T. is an inventor of intellectual property used in the study and may gain royalties from future commercialization of the technology licensed to Converge Biotech Inc. A.A.T. and V.M. are stock option holders in Converge Biotech. F.W.P. and C.T. are full-time employees and stock option holders in Semma Therapeutics, Inc., a wholly owned subsidiary of Vertex Pharmaceuticals Inc. Y.C.P., A.R., and L.Y. were employees of Semma Therapeutics at the time of this work. Y.C.P. and F.W.P. are co-inventors of intellectual property used in this study. C.R. is part of Semma Therapeutics's SAB. The other authors declare no competing interests.

ACKNOWLEDGMENTS

The authors would like to thank Dr. Christopher Fraker, DRI, for his help with OCR experiments and analysis; Dr. Peter Buchwald, DRI, for his help with perifusion assays; Dr. Diana Velluto for providing PEG-OES material for encapsulation; the DRI Small Animal and Transplational Models Core for management of diabetic mice; and the DRI Histology and Analytical Imaging Core for assisting with histological processing of samples, and the DRI cGMP Cell Processing Facility for the isolation of cadaveric islets. Funding was provided by Semma Therapeutics, the Diabetes Research Institute Foundation, and the National Institutes of Health (grant #DK109929).

Received: February 6, 2019
Revised: November 8, 2019
Accepted: November 15, 2019
Published: December 12, 2019

REFERENCES

Abdul-Ghani, M.A., Lyssenko, V., Tuomi, T., Defronzo, R.A., and Groop, L. (2010). The shape of plasma glucose concentration curve during OGTT predicts future risk of type 2 diabetes. Diabetes Metab. Res. Rev. *26*, 280–286.

Allen, C.F.H., and Humphlett, W.J. (1966). The thermal reversibility of the Michael reaction: V. The effect of the structure of certain thiol adducts on cleavage. Can. J. Chem. *44*, 2315–2321.

Atkinson, M.A., Eisenbarth, G.S., and Michels, A.W. (2014). Type 1 diabetes. Lancet *383*, 69–82.

Barkai, U., Rotem, A., and de Vos, P. (2016). Survival of encapsulated islets: more than a membrane story. World J. Transplant. *6*, 69–90.

Berman, D.M., Molano, R.D., Fotino, C., Ulissi, U., Gimeno, J., Mendez, A.J., Kenyon, N.M., Kenyon, N.S., Andrews, D.M., Ricordi, C., et al. (2016). Bioengineering the endocrine pancreas: intraomental islet transplantation within a biologic resorbable scaffold. Diabetes *65*, 1350–1361.

Berney, T., and Johnson, P.R. (2010). Donor pancreata: evolving approaches to organ allocation for whole pancreas versus islet transplantation. Transplantation *90*, 238–243.

Brubaker, C.E., Velluto, D., Demurtas, D., Phelps, E.A., and Hubbell, J.A. (2015). Crystalline oligo(ethylene sulfide) domains define

highly stable supramolecular block copolymer assemblies. ACS Nano *9*, 6872–6881.

Buchwald, P., Tamayo-Garcia, A., Manzoli, V., Tomei, A.A., and Stabler, C.L. (2018). Glucose-stimulated insulin release: parallel perifusion studies of free and hydrogel encapsulated human pancreatic islets. Biotechnol. Bioeng. *115*, 232–245.

Calafiore, R., and Basta, G. (2014). Clinical application of microencapsulated islets: actual prospectives on progress and challenges. Adv. Drug Deliv. Rev. *67-68*, 84–92.

Chen, T., Yuan, J., Duncanson, S., Hibert, M.L., Kodish, B.C., Mylavaganam, G., Maker, M., Li, H., Sremac, M., Santosuosso, M., et al. (2015). Alginate encapsulant incorporating CXCL12 supports long-term allo- and xenoislet transplantation without systemic immune suppression. Am. J. Transplant. *15*, 618–627.

D'Amour, K.A., Bang, A.G., Eliazer, S., Kelly, O.G., Agulnick, A.D., Smart, N.G., Moorman, M.A., Kroon, E., Carpenter, M.K., and Baetge, E.E. (2006). Production of pancreatic hormone-expressing endocrine cells from human embryonic stem cells. Nat. Biotechnol. *24*, 1392–1401.

Dean, P.G., Kukla, A., Stegall, M.D., and Kudva, Y.C. (2017). Pancreas transplantation. BMJ *357*, j1321.

Dionne, K.E., Colton, C.K., and Yarmush, M.L. (1993). Effect of hypoxia on insulin secretion by isolated rat and canine islets of Langerhans. Diabetes *42*, 12–21.

Duvillie, B. (2013). Vascularization of the pancreas: an evolving role from embryogenesis to adulthood. Diabetes *62*, 4004–4005.

Garcia, A.J. (2014). PEG-maleimide hydrogels for protein and cell delivery in regenerative medicine. Ann. Biomed. Eng. *42*, 312–322.

Hering, B.J., Clarke, W.R., Bridges, N.D., Eggerman, T.L., Alejandro, R., Bellin, M.D., Chaloner, K., Czarniecki, C.W., Goldstein, J.S., Hunsicker, L.G., et al. (2016). Phase 3 trial of transplantation of human islets in type 1 diabetes complicated by severe hypoglycemia. Diabetes Care *39*, 1230–1240.

International Diabetes Federation (2017). IDF Diabetes Atlas 8th Edition (IDF). https://www.idf.org/e-library/epidemiology-research/diabetes-atlas/134-idf-diabetes-atlas-8th-edition.html.

Kharkar, P.M., Rehmann, M.S., Skeens, K.M., Maverakis, E., and Kloxin, A.M. (2016). Thiol-ene click hydrogels for therapeutic delivery. ACS Biomater. Sci. Eng. *2*, 165–179.

Korsgren, O. (2017). Islet encapsulation: physiological possibilities and limitations. Diabetes *66*, 1748–1754.

Krishnan, R., Ko, D., Foster, C.E., 3rd, Liu, W., Smink, A.M., de Haan, B., De Vos, P., and Lakey, J.R. (2017). Immunological challenges facing translation of alginate encapsulated porcine islet xenotransplantation to human clinical trials. Methods Mol. Biol. *1479*, 305–333.

Kroon, E., Martinson, L.A., Kadoya, K., Bang, A.G., Kelly, O.G., Eliazer, S., Young, H., Richardson, M., Smart, N.G., Cunningham, J., et al. (2008). Pancreatic endoderm derived from human embryonic stem cells generates glucose-responsive insulin-secreting cells in vivo. Nat. Biotechnol. *26*, 443–452.

Lee, K.Y., and Mooney, D.J. (2012). Alginate: properties and biomedical applications. Prog. Polym. Sci. *37*, 106–126.

Manzoli, V., Villa, C., Bayer, A.L., Morales, L.C., Molano, R.D., Torrente, Y., Ricordi, C., Hubbell, J.A., and Tomei, A.A. (2018). Immunoisolation of murine islet allografts in vascularized sites through conformal coating with polyethylene glycol. Am. J. Transplant. *18*, 590–603.

Markmann, J.F., Bartlett, S.T., Johnson, P., Korsgren, O., Hering, B.J., Scharp, D., Kay, T.W., Bromberg, J., Odorico, J.S., Weir, G.C., et al. (2016). Executive summary of IPITA-TTS opinion leaders report on the future of beta-cell replacement. Transplantation *100*, e25–31.

Merani, S., Truong, W., Emamaullee, J.A., Toso, C., Knudsen, L.B., and Shapiro, A.M. (2008). Liraglutide, a long-acting human glucagon-like peptide 1 analog, improves glucose homeostasis in marginal mass islet transplantation in mice. Endocrinology *149*, 4322–4328.

Millman, J.R., and Pagliuca, F.W. (2017). Autologous pluripotent stem cell-derived beta-like cells for diabetes cellular therapy. Diabetes *66*, 1111–1120.

Millman, J.R., Xie, C., Van Dervort, A., Gurtler, M., Pagliuca, F.W., and Melton, D.A. (2016). Generation of stem cell-derived beta-cells from patients with type 1 diabetes. Nat. Commun. *7*, 11463.

Muthyala, S., Safley, S., Gordan, K., Barber, G., Weber, C., and Sambanis, A. (2017). The effect of hypoxia on free and encapsulated adult porcine islets-an in vitro study. Xenotransplantation *24*. https://doi.org/10.1111/xen.12275.

Nishimura, M., Iizuka, N., Fujita, Y., Sawamoto, O., and Matsumoto, S. (2017). Effects of encapsulated porcine islets on glucose and C-peptide concentrations in diabetic nude mice 6 months after intraperitoneal transplantation. Xenotransplantation *24*. https://doi.org/10.1111/xen.12313.

Nunamaker, E.A., Purcell, E.K., and Kipke, D.R. (2007). In vivo stability and biocompatibility of implanted calcium alginate disks. J. Biomed. Mater. Res. A *83*, 1128–1137.

O'Shea, G.M., Goosen, M.F., and Sun, A.M. (1984). Prolonged survival of transplanted islets of Langerhans encapsulated in a biocompatible membrane. Biochim. Biophys. Acta *804*, 133–136.

Orive, G., Emerich, D., Khademhosseini, A., Matsumoto, S., Hernandez, R.M., Pedraz, J.L., Desai, T., Calafiore, R., and de Vos, P. (2018). Engineering a clinically translatable bioartificial pancreas to treat type I diabetes. Trends Biotechnol. *36*, 445–456.

Pagliuca, F.W., Millman, J.R., Gurtler, M., Segel, M., Van Dervort, A., Ryu, J.H., Peterson, Q.P., Greiner, D., and Melton, D.A. (2014). Generation of functional human pancreatic beta cells in vitro. Cell *159*, 428–439.

Papas, K.K., Avgoustiniatos, E.S., and Suszynski, T.M. (2016). Effect of oxygen supply on the size of implantable islet-containing encapsulation devices. Panminerva Med. *58*, 72–77.

Papas, K.K., Bellin, M.D., Sutherland, D.E., Suszynski, T.M., Kitzmann, J.P., Avgoustiniatos, E.S., Gruessner, A.C., Mueller, K.R., Beilman, G.J., Balamurugan, A.N., et al. (2015). Islet oxygen consumption rate (OCR) dose predicts insulin independence in clinical islet autotransplantation. PLoS One *10*, e0134428.

Qayyum, A.S., Jain, E., Kolar, G., Kim, Y., Sell, S.A., and Zustiak, S.P. (2017). Design of electrohydrodynamic sprayed polyethylene

glycol hydrogel microspheres for cell encapsulation. Biofabrication *9*, 025019.

Redondo, M.J., Steck, A.K., and Pugliese, A. (2018). Genetics of type 1 diabetes. Pediatr. Diabetes *19*, 346–353.

Rodriguez-Diaz, R., Molano, R.D., Weitz, J.R., Abdulreda, M.H., Berman, D.M., Leibiger, B., Leibiger, I.B., Kenyon, N.S., Ricordi, C., Pileggi, A., et al. (2018). Paracrine interactions within the pancreatic islet determine the glycemic set point. Cell Metab. *27*, 549–558.e4.

Safley, S.A., Kenyon, N.S., Berman, D.M., Barber, G.F., Willman, M., Duncanson, S., Iwakoshi, N., Holdcraft, R., Gazda, L., Thompson, P., et al. (2018). Microencapsulated adult porcine islets transplanted intraperitoneally in streptozotocin-diabetic non-human primates. Xenotransplantation *25*, e12450.

Salama, B.F., and Korbutt, G.S. (2017). Porcine islet xenografts: a clinical source of ss-cell grafts. Curr. Diab. Rep. *17*, 14.

Scharp, D.W., and Marchetti, P. (2014). Encapsulated islets for diabetes therapy: history, current progress, and critical issues requiring solution. Adv. Drug Deliv. Rev. *67-68*, 35–73.

Schneider, S., Feilen, P.J., Brunnenmeier, F., Minnemann, T., Zimmermann, H., Zimmermann, U., and Weber, M.M. (2005). Long-term graft function of adult rat and human islets encapsulated in novel alginate-based microcapsules after transplantation in immunocompetent diabetic mice. Diabetes *54*, 687–693.

Schuetz, C., and Markmann, J.F. (2016). Islet cell transplant: update on current clinical trials. Curr. Transplant. Rep. *3*, 254–263.

Shapiro, A.M., Pokrywczynska, M., and Ricordi, C. (2017). Clinical pancreatic islet transplantation. Nat. Rev. Endocrinol. *13*, 268–277.

Shimoda, M., and Matsumoto, S. (2017). Microencapsulation in clinical islet xenotransplantation. Methods Mol. Biol. *1479*, 335–345.

Skyler, J.S. (2018). Hope vs hype: where are we in type 1 diabetes? Diabetologia *61*, 509–516.

Smith, K.E., Kelly, A.C., Min, C.G., Weber, C.S., McCarthy, F.M., Steyn, L.V., Badarinarayana, V., Stanton, J.B., Kitzmann, J.P., Strop, P., et al. (2017). Acute ischemia induced by high-density culture increases cytokine expression and diminishes the function and viability of highly purified human islets of langerhans. Transplantation *101*, 2705–2712.

Stegall, M.D., Lafferty, K.J., Kam, I., and Gill, R.G. (1996). Evidence of recurrent autoimmunity in human allogeneic islet transplantation. Transplantation *61*, 1272–1274.

Tomei, A.A., Manzoli, V., Fraker, C.A., Giraldo, J., Velluto, D., Najjar, M., Pileggi, A., Molano, R.D., Ricordi, C., Stabler, C.L., et al. (2014). Device design and materials optimization of conformal coating for islets of Langerhans. Proc. Natl. Acad. Sci. U S A *111*, 10514–10519.

Tomei, A.A., Villa, C., and Ricordi, C. (2015). Development of an encapsulated stem cell-based therapy for diabetes. Expert Opin. Biol. Ther. *15*, 1321–1336.

Vaithilingam, V., Bal, S., and Tuch, B.E. (2017). Encapsulated islet transplantation: where do we stand? The review of diabetic studies. Rev. Diabet. Stud. *14*, 51–78.

Vegas, A.J., Veiseh, O., Gurtler, M., Millman, J.R., Pagliuca, F.W., Bader, A.R., Doloff, J.C., Li, J., Chen, M., Olejnik, K., et al. (2016). Long-term glycemic control using polymer-encapsulated human stem cell-derived beta cells in immune-competent mice. Nat. Med. *22*, 306–311.

Veres, A., Faust, A.L., Bushnell, H.L., Engquist, E.N., Kenty, J.H., Harb, G., Poh, Y.C., Sintov, E., Gurtler, M., Pagliuca, F.W., et al. (2019). Charting cellular identity during human in vitro beta-cell differentiation. Nature *569*, 368–373.

Verheyen, C.A., Morales, L., Sussman, J., Paunovska, K., Manzoli, V., Ziebarth, N.M., and Tomei, A.A. (2019). Characterization of polyethylene glycol–reinforced alginate microcapsules for mechanically stable cell immunoisolation. Macromol. Mater. Eng. *304*. https://doi.org/10.1002/mame.201800679.

Villa, C., Manzoli, V., Abreu, M.M., Verheyen, C.A., Seskin, M., Najjar, M., Molano, R.D., Torrente, Y., Ricordi, C., and Tomei, A.A. (2017). Effects of composition of alginate-polyethylene glycol microcapsules and transplant site on encapsulated islet graft outcomes in mice. Transplantation *101*, 1025–1035.

Stem Cell Reports, Volume *14*

Supplemental Information

Conformal Coating of Stem Cell-Derived Islets for β Cell Replacement in Type 1 Diabetes

Aaron A. Stock, Vita Manzoli, Teresa De Toni, Maria M. Abreu, Yeh-Chuin Poh, Lillian Ye, Adam Roose, Felicia W. Pagliuca, Chris Thanos, Camillo Ricordi, and Alice A. Tomei

Supplemental Figure and legend

Figure S1:

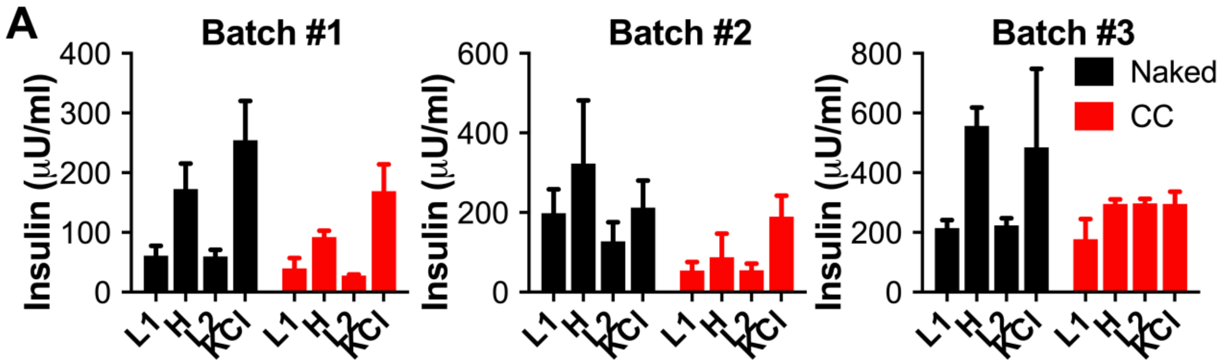

Figure S1: *In vitro* GSIS assessment of unencapsulated and CC SC-islets

(A) GSIS functionality of three independent batches of unencapsulated (black, naked) and conformal coated (red, CC) SC-islets 48 hours (S6d11) after encapsulation as absolute insulin secretion (n=3 wells assayed per condition) during sequential stimulation with 2.8mM glucose (L), 20mM glucose (H), 2.8mM glucose (L), and 30mM KCl.
